# The methodology wars and outdoor and environmental education: Feminism, positivism, and causation

**DOI:** 10.1007/s42322-022-00103-3

**Published:** 2022-08-04

**Authors:** Jack Reed

**Affiliations:** Moray House School of Education and Sport, The University of Edinburgh, Holyrood Road, EH8 8AQ Edinburgh, Scotland

**Keywords:** Causation, Methodology wars, Feminism, Positivism

## Abstract

How causation is approached has, for some time now, been a central debate within the archives of educational research. Despite rich discussion in broader literature, the influence of what has been described as the ‘methodology wars’ has rarely featured within the field(s) of outdoor and environmental education (OEE). This paper explores causation in this context, employing a feminist paradigmatic approach to investigate the role of causation in OEE research. A positivist approach is also considered in parallel, asking whether and how research in OEE navigates causation, and the potential influences of this upon competing audiences (e.g., policy makers and funders). Drawing on a *conceptual causal pluralist* approach to causation within the feminist paradigm, four key touchstones are presented that stand ready to facilitate inclusive, equitable, and reflexive research for OEE post-pandemic. The paper reflects on the general position of OEE presently, and responds to increasing sociocultural complexity as it is lived and felt within the profession and beyond.

## Introduction

The relationship(s) between broader educational inquiry and research that seeks to expose causal relationships has, for some time, been a significant topic for scholarly debate outwith the outdoor and environmental education (OEE) literature. As discussed by Rowbottom and Aiston (2006), to what extent the scientific method, or clinical trial, features in educational research has been a repetitive issue in its history. Whilst the writings of Poplin (1987), Matthews (2004), and Mackenzie (2011) offer a welcome set of exchanges on the matter, which was reignited recently by Thomas (2021), it is perhaps the dialogues presented in volume 21 of the journal *Educational Researcher* that contextualises the question: how does research in education expose causal relationships, and why does it matter? Taking the form of a discussion, Schrag (1992a; 1992b), Eisner (1992), and Erickson (1992) debate the merits and alternatives of a positivistic approach in educational research. For Schrag (1992a), any educational research that addresses questions centred on a causal hypothesis will have to commit itself to an educational trial. Alternative research approaches may derive new practices, policies, or ways of thinking, but without the educational trial these claims can be considered unreliable or fallacious. Meanwhile, Eisner (1992) and Erickson (1992) refute these claims. They question whether a universal approach to understanding reality, which draws on a foundationalist conception of knowledge and disconnects value from fact, oversimplifies the situated experiences of real-world teaching and learning where a single, universal, semblance of reality cannot exist. As Alexander (2006) neatly put it, this dichotomy may be called the “methodology wars” (p. 206). Over two decades ago, Allison and Pomeroy (2000) explored this in the experiential education context, and stated that the field needs to explore a range of paradigms to uncover the situated and complex nature of the processes involved in outdoor learning.

This discourse warrants closer examination within the fields of contemporary OEE and outdoor learning to assess the place and relevance of research that exposes causal relationships. Such a discussion comes at an important time for the field in the context of COVID-19 where across the planet we have seen OEE often relegated and, in some cases, halted altogether (Quay et al., 2020). The question remains, in relation to capturing and sharing the well-acknowledged benefits of learning outdoors (e.g., James & Williams 2017; Schwass et al., 2021), what role does causation play in the development and realisation of research? And, as I discuss in this paper, what role might our ontological and epistemological assumptions play in the development of inclusive and equitable research? This paper could reflect on many paradigms to assess the place of OEE in the so-called methodology wars. However, drawing on work (Ackerly & True, 2020; Cosgrove & McHugh, 2000; Daley, 2010) that recognises the importance of how knowledge, power, relationships, and context affect the research process, this paper considers whether attentiveness to epistemic belief may alter the ways in which causal relationships are interpreted and defined in OEE.

To do this, a feminist approach is employed that, as discussed by Hesse-Biber (2012), provides a platform from where a range of epistemologies and methodologies may be interrogated and transformed. This interrogation is of critical importance when acknowledging and celebrating research processes as chaotic, non-linear, and heterogeneous. To offer a critical view, positivism is also discussed as a lens through which the feminist standpoint might be challenged or reinforced. The paper concludes that, in OEE, research *does* expose causal relationships so long as we reframe our understanding of ‘causality’ to encompass and extend beyond the scientific method. Within this, four touchstones are introduced that jointly identify how causation can take many forms and, perhaps crucially, that recognise a need for causal approaches that stand ready to address the complexities of 21st century OEE. In addressing these complex issues, I turn to the work of de Sousa Santos (2014) on the *Sociology of Absences* and to Smith’s (2021) work on *Māori feminist research approaches* to explore their need and relevance within the field of OEE. I begin by providing initial definitions of what I mean by ‘causal relationships’, as well as operationally defining feminism and positivism. I then turn to OEE literature, exploring causation in practice, and, finally, assess whether an explicit understanding of an alternative frame of causation may be useful as OEE recovers from COVID-19.

Before going any further, I have grappled with my position as a white heterosexual male in this field and continuously asked myself whether I am the right person to write a paper on the relationship(s) between feminism, positivism, and causation. Unconscious bias, unacknowledged power, and privilege were just some of the factors that came to mind. The truth is I am not sure I am the right person to compose a manuscript on the intersections of causation within feminism and positivism in OEE. Was this a reason to discontinue the project? I turned to Tienari and Taylor (2019), two men who have written about their interpretations, vulnerabilities, and positions within feminism, to help me understand my position and my contribution. I was taken by their discussion on the potentials and importance of men engaging with cultures of exclusion, domination, and prejudice. I also reflected on the texts of Van der Gaag (2014) and Smolović-Jones et al. (2021) and concluded that if we (as practitioners and researchers) want to make a lasting and inclusive difference in OEE, then irrespective of gender we have a duty to do so. This acknowledges that a feminist paradigmatic approach to causation is too important to shelve on account of my position as a white heterosexual male. I therefore proceed with the upmost reflexivity as I present the tensions between feminism, positivism, and causation in OEE.

### What are ‘causal relationships’ in research?

The traditional view of causal relationships in research, that is the cause and effect relationship whereby a cause variable (X) leads to changes in an outcome variable (Y), has provided “the basis of ordinary quantitative [and experimental] research” (Mohr, 1996, p. 99). For Morris et al. (2016), one such approach for uncovering causal relationships in education is the use of randomised controlled trials. However, to what extent the randomised controlled trial in OEE captures, for instance, on the ground lived experiences remains unclear. This leads me to Reed’s (2011) ‘landscapes of meaning’ which acknowledges that the researcher’s primordial task is to paint the landscape of the phenomena under investigation, thus enabling the meanings of historical, social, and cultural actions to be discovered and shared. This indicates that causal relationships are discoverable in both qualitative and quantitative research, but are interpreted and understood based on structures of epistemic perspective that are framed by interconnected social, cultural, and historical factors. Nichols’s (2000) research agenda for adventure education ‘gets at’ some of this complexity when discussing the classical experimental causality model as impractical, and that it *may* hold an unrealistic view of the social world in outdoor learning. Further, Warren and Loeffler’s (2000) paper on social justice research in outdoor experiential education holds resonance. They suggest there is an absence of developed approaches to research that stand ready to emancipate the voices and experiences of marginalised groups. As I discuss later in the paper, the ways in which we are attentive to our ontological and epistemic beliefs are important when developing reflexive approaches to research in OEE that do not inopportunely overlook or discount the experiences of disempowered voices.

### Introducing feminism and positivism: mapping the nature of causation

#### Feminism

Hooks (2000) acknowledged that there has been a lack of consensus on a definition of feminism due to its diversity and wide-ranging purposes. From the work of Wittgenstein (1953/2009), feminism can be considered a broad family of critical approaches which question the status quo, counter oppression, and has at its core the recognition that gender/sex must be examined when attempting to evaluate the social world. Drawing on the literature cited by Hesse-Biber (2012, p. 3-4), a feminist approach is attentive to structures of authority and power in research that extend beyond androcentric bias. Feminism empowers the researcher to see and embrace diversity, whilst overtly acknowledging and challenging intersecting discriminatory structures which serve to subjugate how knowledge is produced. As Dillard (2000) outlined, the beating heart of feminism re-frames the research endeavour as one that liberates the marginalised and brings into question “the traditions, perspectives, viewpoints, cultural understandings, and discourse style of the researcher” (p. 663). I do not have to look far in the OEE literature to acknowledge the salience of feminism in practice (Allin & West, 2013; Bren & Prince, 2022; Gray, 2016; Haq et al., 2020; O’Brien & Allin, 2021; Wall, 2017; Warren & Rheingold, 1996) and the continued oppression, misogyny, and sexual harassment experienced by diverse groups of people in the outdoors (Davies et al., 2019; Gray et al., 2020; Kennedy & Russell, 2020; Warren et al., 2018). That said, when seeking research with an explicit feminist paradigmatic approach in OEE, the results often yield limited applications. Although work such as Lynch et al. (2020) explicitly discusses feminist approaches to data collection, there appears to have been a limited ‘banging of the drum’ on the role of an explicit feminist paradigm for the field.

It is therefore important to map the nature of causation in feminist research. Drawing on Crasnow’s (2015) chapter on causal pluralism in feminist research, this paper now asks: can a research approach focussed on emancipative processes expose causal relationships in OEE? Whilst many have discussed plurality in causality (e.g., Campaner & Galavotti 2007; De Vreese, 2006; Weber, 2007), Crasnow (2015) defines causal pluralism as “the view that there is more than one form [of] causal explanation” (p. 637). In essence, in order to fully understand the situated complexity of individual experiences, the positivist focus on ‘average effect causation’ is called into question. Cartwright (1999, 2004) picked up on this, arguing that applying universalism to a cause and effect relationship cannot identify which individual features of the cause and effect are both felt and realised at the individual level. However, whilst Cartwright (2006) agreed an alternative theory of causal relationships was necessary, they showed that “we do not have any theories [of causal plurality] at all” (p. 66). Both Reiss (2011) and Crasnow (2011, 2015) picked up on this. Whilst Reiss (2011) discussed wide-ranging theoretical approaches to pluralism, including evidential and metaphysical causal pluralism, Crasnow (2015) outlined a *conceptual pluralism* for understanding causal relationships in feminist research. Put simply, conceptual causal pluralism places the very notion of ‘cause’ under scrutiny. By positing that ‘cause’ will have different effects within a diverse population, exposing causal relationships in feminist research moves beyond unitary average effect causation that is thought to treat causal relationships as one-dimensional and oppressive for populations at the margin.

#### Positivism

Positivism, broadly framed, is more readily defined in the literature (e.g., Bryant 1985; Giddens, 1974; Neuman, 2014) than its feminist counterpart. Its central argument recognises that, through experimentation and the scientific method, the natural and social world can be understood and improved by employing deductive reasoning and precise empirical scrutiny. Whilst Blaikie and Priest (2017) sketch out the origins of positivism, including reference to Comte (1830/1988), Durkheim (1895/1938), and Hume (1888), the basis of positivism in this paper is drawn from Howe (2009) and Mackenzie (2011). Together, they state that positivism, grounded in philosophy and characterised by the scientific method, is a bias-neutral paradigm which seeks to uncover nomothetic knowledge through exact empirical inquiry by developing understanding in the form of universal laws. Turning to literature in OEE, which I take to constitute empirical and peer-reviewed research within outdoor learning contexts, it is clear that quantitative approaches have been readily employed in numerous studies (e.g., Cooley et al., 2016; Stott & Hall, 2003; Scrutton, 2015, 2020). However, it is Scrutton and Beames’s (2015) paper that overarchingly discusses quantitative approaches for outdoor learning, and specifically its applicability for research seeking to assess personal and social development outcomes within the context of outdoor and adventurous education. Alongside recognising the importance of statistical evidence for stakeholders, their analysis of 28 quantitatively grounded papers revealed a series of methodological limitations that restrict operational rigour for studies within the positivist paradigm. This raises important issues for the field of OEE. If we seek to publish research that evaluates situated experience and complexity, then how does this paradigmatically and practically match stakeholder expectation, the accessibility of future funding, and the position of OEE in broader curricula endeavours? This question ultimately links back to Allison and Pomeroy’s (2000) core question, that is, “how shall we know?” (p. 92), and I add, how shall we share?

Gerring (2005) acknowledges that the traditional, *unitary*, view of causation has come under increasing scrutiny in the social sciences. Despite the increased scrutiny, Checkel (2006) recognises positivism’s theory of unitary causation as the foundational scientific measure which “provides the how-we-come-to-know nuts and bolts for mechanism-based accounts of social change” (p. 363). In relation to causation, then, the positivist approach enables causal relationships to be exposed, explained, and predicted at an empirically large scale whilst being replicable (Park et al., 2020). As Freedman (2006) describes, positivistic approaches to exposing causal relationships “offer more reliable evidence on causation than observational studies” (p. 691), which are thought to lack broad applicability across society. It is this which contributed to the United Kingdom’s Department for Education (2018) recognising positivism as the primary approach from which to base its research agenda and policy development; for examples, see Department for Education (2019, 2020a, 2020b). One significant benefit to the Department for Education’s approach is expressed by Hargreaves (1997) and Gorard et al. (2017), that is the ability to assess and implement *evidence-based practice*. According to Thyer’s (2008) paper, which is grounded in social work, liberal views and caring attitudes will not expose causal relationships that can readily influence practice. Instead, they state that if we are to seek “the best available evidence” (p. 344) which exposes causal relationships for practice development, then we will ultimately have to “embrace the fundamental tenets of positivism” (p. 339).

## Through the looking-glass: shifting conceptions of causal relationships in outdoor and environmental education research

### A rejoinder: confusion, complexity, or causation?

Whilst Gerring (2005) and Crasnow (2015) both approach the notion of causation from different standpoints, both acknowledge the term ‘causal relationships’ to be ironically polysemantic. Indeed, despite the contrast described between positivist and feminist approaches to causation, Leckenby (2007) outlines a feminist empiricist approach that they identify as unwaveringly positivistic yet underpinned by feminist values and criticality. To get to the bottom of this tension in OEE research, it is worth considering what Allin and Humberstone (2006) and Quay (2016) discuss on the social, cultural, and environmental *complexities* which encompass educating young people out-of-doors. We know that there are innumerable intersecting factors that frame pedagogical practice and the experiences of participants. As Christie et al. (2016) note when quoting Davis and Sumara’s (2006, p. xi) work on complexity in education, outdoor learning endeavours are so complex and heterogenous they surely defy “simplistic analyses and cause-effect explanations” (p. xi). Reason for this is found in the work of Byrne (2005) who claims the positivist view on causation relies too much on linearity. Acknowledging the complexity of social life therefore depends on recognising that there is an assemblage of multiple, interrelated, and unpredictable causes that frame the nature of reality. To what ends, then, can linear and controlled approaches to causation fully explicate the nature of complex and fluid human lives? This raises an important consideration surrounding the efficacy of paradigmatic standpoints within OEE and whether they stand ready to capture and share the inherent complexities and nuance that naturally emerge when educating people out-of-doors.

Is that the end of that then? Positivism can expose causal relationships in OEE, but cannot fully capture the complexity of experience, learning, and growth when learning outside? Recent work that has employed a positivist ‘average effect’ causal approach in outdoor learning (e.g., Beames et al., 2018; Cooley et al., 2020; Scrutton, 2020) suggests this view is too simple an answer. Quibell et al. (2017) somewhat alleviate this causal tension by outlining *why* the unitary view on exposing causal relationships is important in outdoor learning. They acknowledge how, without substantial and conclusive statistical evidence, the benefits of learning outdoors cannot be captured and shared with policy makers and curriculum developers in a meaningful and accessible way. This is of added importance when considering the work of Quay et al. (2020) on the negative impacts the pandemic has had on OEE. There has been a real risk that OEE could be considered an expendable commodity in the education of young people. What is required, then, could be a blending of both Gerring’s (2005) and Crasnow’s (2015) assessments of causation if OEE is to remain a viable endeavour in the eyes of policy makers and funders. However, whilst it can be claimed that both feminist and positivist approaches to causation have merit in the field of OEE, it is worth acknowledging that adventurous activities and outdoor environments have been considered an inherently male space (Gray, 2016; Kennedy & Russell, 2020). This points us towards Brooks’s (2007) outlining of what they call the ‘dominant knowledge canons’ in research that naturally questions whether employing unitary average effects in OEE may inopportunely expose causal relationships from the more dominant male standpoint only. It is this that returns me to Leckenby’s (2007) feminist empiricist approach that could unlock the potential of positivism for OEE whilst remaining critically attentive to all voices and perspectives.

#### The challenge

To bring to life the purpose of the feminist paradigm in OEE, I draw on Marshall (1994), Parsons and Priola (2013), and Berila (2021), to recognise feminist research as a mediator for social transformation. To return to Ackerly and True (2020), the purpose of good feminist research is to “push the boundaries of how other scholars have understood things” (p. 258). Centrally, they acknowledge that feminist research is above all *attentive*; attentive to the power of epistemic belief but also attentive to boundaries and relationships in the research process. However, before social transformation can be assessed as an alternative conception of causal relationships, Mishra’s (2013) and Todd’s (2016) problematisation of what has been outlined above requires acknowledgement. Both recognise traditional feminist research to normalise feminism’s purpose from a Western perspective, which may marginalise indigenous, racial, and ethnic minority populations. Both authors call for a postcolonial or indigenous feminist approach to research that allow the transformative purposes of feminism to be realised. This turns me to Smith’s (2021) work and their citing of de Sousa Santos (2014) and, specifically, de Sousa Santos’s theorising on the *Sociology of Absences*. The Sociology of Absences is important for this discussion as it does not begin by asking ‘what is present?’, as we may in traditional research approaches, but begins by asking what is functionally produced, in terms of knowledge, as null, non-existent, or unseen? As de Sousa Santos (2014) explains, a Sociology of Absences pulls into question “the positivistic principle that consists of reducing reality to what exists and to what can be analyzed with the methodological and analytical instruments of the conventional social sciences” (p. 172). It is thus claimed that by focussing on what is absent and what is assumed within the development, realisation, and sharing of research that we may address what de Sousa Santos (2014) defines as the centrality of Westernised monocultural knowledge. As Smith (2021) explains, this may reinvigorate the purpose and scope of research, as research which focusses on social justice “is an intellectual, cognitive and moral project, often fraught, never complete, but worthwhile” (p. 270).

#### So, does the ‘best’ research expose causal relationships?

The advantages of incorporating plurality in the exposure of causal relationships from both feminist and positivist standpoints have now been discussed. This, alongside questioning what is absent and what is present in traditional discourses of knowledge, generates challenging yet necessary terrain for research in OEE to navigate. Focussing on Shaheen (2016), it is, however, noted that causal plurality is at risk of developing causal ambiguity, where any cause and effect may falsely be claimed an exposure of a causal relationship. It is therefore important to ask exactly how causal plurality fits within OEE research. When operationally defining what we may describe as ‘best’ research, that is how a chosen research design can effectively answer the chosen research question, it is reasonable to suggest that the overarching purpose of research is to expose causal relationships. However, if best research is to fully evaluate the complexities of the social world, then re-applying epidemiology’s metaphor of the *web of causation* (Venkatapuram, 2011) to the social sciences could be useful for OEE. As Ventriglio et al. (2016) outline, the web of causation posits that multiple causative relationships often exist and influence an effect at any one time and therefore require multiple research approaches to fully uncover them. Weber et al. (2005) reaffirm this through their acknowledgement that cause and effect relationships are often structured and discovered based on both research design and the reflexivity of the researcher. It is this diversity which enables new research questions to be explored, research questions that can reframe how causation is interpreted, such as employing Crasnow’s (2015) conceptual causal pluralism, to liberate previously unrecognised causal relationships within oppressed and marginalised populations. In so doing, questions of what is absent and what is decided (consciously or subconsciously) to be non-existent may come to the fore and be considered. Whilst doing this, research must be attentive to Ackerly and True’s (2020) feminist research ethic that ensures the situatedness of the researcher is acknowledged and mitigated. Ultimately, by recognising the interplay between research design and research question, research that ensures all voices are heard and all perspectives are accounted for represents an exemplary benchmark for research in OEE.

That being said, dismissing positivism as secondary research and as the vanguard against inclusion and alternative conceptions of causation risks “throwing … the baby out with the bathwater” (Husén, 1988, p. 13). It is this that leads me to question Howe’s (2004) assertion that the positivist/non-positivist debate in educational research is a matter of value-based duality; right and wrong, left and right. Instead, drawing on Lather (2006), approaching research and causation from a standpoint of paradigmatic diversity could allow a multitude of perspectives to come to the fore that can more readily assess the “complexities of language and the world” (p. 36). What we are at risk of here, however, is discussed by Halliday (2002) who, when citing Griffiths (1998), stated that “sitting on the [paradigmatic] fence is unproductive and probably impossible” (p. 53). The best research cannot be considered to expose causal relationships when ambiguity and methodological uncertainty frame the research process. To understand how we can arrive at best research, which mutually recognises the historical and contemporary purposes of both the feminist and positivist perspectives, returning to Smith’s (2021) work on the decolonisation of epistemology and methodology offers paradigmatic hope, and offers opportunity to re-evaluate how we design and implement research in OEE. As Wilson (2001), Mishra (2013), and Todd (2016) discuss, how can we arrive at the claim that best research exposes causal relationships without acknowledging the colonial backdrop to such a statement? Smith (2021) shows how what can be considered best research in the paper thus far needs to ‘research back’ to disrupt the rules and practices of traditional research. By utilising a Māori feminist approach, Smith (2021) argues that if research is to expose causal relationships in a manner that does not oppress or marginalise, research must focus on developing culturally appropriate ontologies, epistemologies, and methodologies. What emerges, then, are key touchstones for research that must be evaluated in OEE. These are: recognising causation may take many forms; remaining attentive to the situated complexity of the social world and people’s experiences of it; acknowledging and addressing how ontology, epistemology, and research approaches can inopportunely marginalise populations; and finally, embracing the research process as non-linear and heterogeneous. When incorporating these four touchstones, OEE researchers can step through the looking-glass and see best research not as something abstract and untouchable, but as something that is ready to embrace the challenges and hurdles that will come with researching learning outside the classroom in the 21st century.

## Implications

These four touchstones have clear implications for future OEE research that must situate itself in what is an increasingly transcultural and globalised world. As Cochran-Smith (2004) and O’Leary (2017) note, a research question that is investigated through different research approaches will likely arrive at different answers. This paper suggests that to fully realise the complexity of the social world in OEE, this diversity in approaches is to be embraced and not prohibited. In addition, Crasnow’s (2015) conceptual causal plurality offers an opportunity to expose causal relationships beyond the confines of one-dimensional interpretations of causation. This view is consolidated when re-applying epidemiology’s metaphor of the *web of causation* (Venkatapuram, 2011) to the social sciences and when considering the importance of a Sociology of Absences (de Sousa Santos, 2014). For OEE, then, how research ensures the historically, socially, culturally, and politically entangled webs of causation are acknowledged and reported is of upmost importance. Critically, this reminds us that, when approaching the plural web of causation, the works of Hesse-Biber (2012), Ackerly and True (2020), and Smith (2021) should be applied to ensure equity and emancipation can come to the fore. This perspective reconsiders a significant question outlined by Preissle and Han (2012), that is, what *could* a feminist research ethic offer us? The implication in this paper encourages us to extend beyond this to ask, what are the consequences if we do *not* employ a feminist perspective in our research?

## Conclusions

Having started by outlining what Alexander (2006) described as the “methodology wars” (p. 206), this paper has assessed the place and role of causation from feminist (e.g., Berila 2021; Parsons & Priola, 2013) and positivist (e.g., Howe 2009; Mackenzie, 2011) perspectives in OEE research. We have seen that getting to the bottom of causation and the purposes of exposing causal relationships are inherently complex and fraught with paradigmatic and methodological hurdles. We have seen how different research approaches will likely answer research questions differently (O’Leary, 2017), how the complexities of experience and policy development in OEE require multiple research approaches to assess causation (Christie et al., 2016; Quibell et al., 2017), and how Crasnow’s (2015) conceptual causal pluralism provides a means through which to do this. What has emerged recognises the importance of Ackerly and True’s (2020) feminist research ethic and, when approaching research in OEE, how this can emancipate multiple forms and interpretations of causation. However, a degree of critical hesitancy is required when considering the works of de Sousa Santos (2014) and Smith (2021). It was questioned whether colonialism provided the cultural backdrop to our quest to assess the methodology wars in OEE. Four key touchstones were therefore outlined with the aim of recentring the research process to recognise how those marginalised and oppressed by the ‘dominant knowledge canons’ (Brooks, 2007) can be heard. What has been discussed recognises that research which does expose causal relationships are situated within layers of sociocultural complexity and the historical suppression of marginalised voices. It is only when this is addressed that research in OEE can enter a new realm of possibility, that is the chance to jointly emancipate causal relationships and marginalised populations. It is this stance that will enable the field to ‘keep up’ with what Schaeffer (2014) describes as the rising tide of global social change.

## Data Availability

N/A

## Code Availability

N/A
